# The Evolutionary History and Functional Divergence of Trehalase (*treh*) Genes in Insects

**DOI:** 10.3389/fphys.2019.00062

**Published:** 2019-02-15

**Authors:** Andrea Nardelli, Matteo Vecchi, Mauro Mandrioli, Gian Carlo Manicardi

**Affiliations:** ^1^Department of Life Sciences, University of Modena and Reggio Emilia, Modena, Italy; ^2^Padiglione Besta, University of Modena and Reggio Emilia, Reggio Emilia, Italy

**Keywords:** trehalase, acid trehalase, gene family evolution, duplicated gene functionalization, insects, phytophagous

## Abstract

Trehalases (*treh*) have been found in different organisms, such as bacteria, fungi, yeast, nematodes, insects, vertebrates, and plants. Their biochemical properties are extremely variable and not yet fully understood. Gene expression patterns have shown differences among insect species suggesting a potential functional diversification of trehalase enzymes during their evolution. A second gene family encoding for enzymes with hypothetical trehalase activity has been repeatedly annotated in insect genome as acid trehalases/acid trehalase-like (*ath*), but its functional role is still not clear. The currently available large amount of genomic data from many insect species may enable a better understanding of the evolutionary history, phylogenetic relationships and possible roles of trehalase encoding genes in this taxon. The aim of the present study is to infer the evolutionary history of trehalases and acid trehalase genes in insects and analyze the trehalase functional divergence during their evolution, combining phylogenetic and genomic synteny/colinearity analyses.

## Introduction

Trehalases, commonly found in different organisms (such as bacteria, fungi, yeast, nematodes, insects, vertebrates, and plants) (Jorge et al., 1997), catalyze the hydrolysis of trehalose into two glucose molecules (Becker et al., 1996). In insects, trehalases have been shown to exist as a soluble form (tre-1) and as a membrane bound form (tre-2) and they play a pivotal role in energy metabolism, chitin biosynthesis, flight and in many other physiological processes, including development and reproduction (Candy, 1974; Shukla et al., 2014). Combining the different role to the variable biochemical properties of trehalases it has been suggested that a functional diversification of trehalase enzymes occurred during insect evolution (Kalf and Rieder, 1958; Duve, 1972; Terra et al., 1978; Huang et al., 2006; Tang et al., 2008, 2012; Chen et al., 2010; Santos et al., 2012.

At present, the availability of a large set of genomic data from many insect species (Yin et al., 2015) makes possible genomic analyses suggesting that: (i) *treh* genes frequently experienced gene duplication during insect evolution; (ii) duplicated copies (paralogs) have been retained in several species bringing to *treh* gene family (Kalf and Rieder, 1958; Duve, 1972; Huang et al., 2006; Tang et al., 2008, 2012; Chen et al., 2010; Santos et al., 2012; Adhav et al., 2018).

Furthermore, the presence of genes coding for acid trehalases/acid trehalase-like (*ath*) has been evidenced in different insects, such as *Bombyx mori* (Lepidoptera: Bombycidae) (Mita, 2004) and *Plutella xylostella* (Lepidoptera: Plutellidae) (You et al., 2013). In other genomes, *ath*/*ath*-like sequences are present as well, but not annotated, as in aphid species *Acyrthosiphon pisum* (Hemiptera: Aphididae; Genome Sequence of the pea aphid *A. pisum*, 2010), *Diuraphis noxia* (Hemiptera: Aphididae) (Nicholson et al., 2015) and *Myzus persicae* (Hemiptera: Aphididae) (Ramsey et al., 2007). Acid trehalases are mainly described in bacteria and fungi (Destruelle et al., 1995; D’Enfert and Fontaine, 1997; Inagaki et al., 2001; Murata et al., 2001) and the origin of such genes in insect genomes is still unexplained.

In view of the consideration that progresses in understanding the molecular characterization of trehalase could favor the use of these proteins as a novel target for insecticides, in the present study we analyzed the evolutionary history of the *treh*/*treh*-like and *ath*/*ath*-like genes in insects with particular emphasis on phytophagous species, combining genomic analyses (related to the sequence presence/conservation and to synteny and/or co-linearity) to the phylogenetic distribution of the observed gene duplications.

## Materials and Methods

The study of *treh/treh-like* and *ath/ath-like* gene families has been performed looking at 40 insect species representatives of five different orders. For each species, genes coding for trehalase, trehalase-like, acid trehalase and acid trehalase-like genes have been identified by retrieving the available coding sequences (even if it was not previously annotated) from NCBI online databases^1^. When trehalase genes were not annotated, transcript sequence from the phylogenetically nearest species has been used to find trehalase genes in the genome of the target species, i.e., *Drosophila pseudoobscura* (Diptera, Drosophilidae) using annotated *D. melanogaster treh* gene. *M. persicae* sequences were retrieved from *Aphidbase* databases^2^.

Nucleotide sequences of exons from NCBI predictions have been used to build *treh* and *ath* gene family phylogenies. For the construction of the trehalase phylogenetic tree, *treh/treh*-like genes sequences from different ecdysozoans other than insects were also analyzed, including the nematode *Caenorhabiditis elegans* (Nematoda: Rhabditidae), the tardigrad *Ramazzottius varieornatus* (Tardigrada, Hypsibiidae) and the crustacean *Artemia franciscana* (Crustacea: Branchiopoda).

Exons sequences were analyzed with *Virtual Ribosome* (Wernersson, 2006) and the predicted coding sequences were aligned with *Muscle* in *MEGA5* (Tamura et al., 2007). The alignment was trimmed to contain only the region between the first and the last conserved domains: VIVPGGR, QWDYPNAWPP, DSKTFVDM, RSQPPL, PRPESYREDY, and ELKAA and glycine rich domain GGGEYE (Barraza and Sánchez, 2013; Xie et al., 2013). The Maximum Likelihood phylogenetic tree was constructed with *raxmlGUI* (Silvestro and Michalak, 2011) (ML + throught bootstrap, 10 runs, 1000 reps, jModeltest and GTRGAMMAI, outgroup *Escherichia coli treh* EU893513.1). The phylogenetic tree was visualized and edited in *iTOL* (Letunic and Bork, 2006). For each conserved domain in each sequence, the *p*-distance with the protein consensus sequence was computed and visualized on the tree to show the level of conservation of the different trehalase genes.

Since trehalase enzymes have been described in insects in two distinct forms (soluble and membrane bound) (i.e., Gu et al., 2009), the amino-acid sequences have been analyzed with *Signalp 4.1* (Petersen et al., 2011) to predict the presence of signal peptides and *TMHMM Server v. 2.0* to predict transmembrane domains (Sonnhammer et al., 1998).

Acid trehalases isoforms have been compared considering the distribution of six consensus discrete motifs, besides the catalytic domain: a transmembrane span (LFFFFFFFLCFSFTTSML), a cAMP-dependent phosphorylation site (RRXS), an EF-like Ca^2+^-binding motif (DTXGDXQITIXD), two trehalase signature motifs 1 and 2, (PGGRFXEXYXWDXY) and (QWDXPX[G/A]W[P/A/S]P), respectively, and the glycosyl phosphatidyl inositol (GPI) membrane anchor motif (CRTNYGYSAA) (Barraza and Sánchez, 2013).

Genomic scaffolds containing *treh* and *ath* genes were compared looking for synteny and co-linearity among insect species by analysis of the neighboring genes located in the same scaffold/contigs hosting *treh* and *ath* genes.

## Results

### Identification of *treh* and *ath* Genes Currently Available in DNA Databases

The search of Genbank databases allowed us to identify 160 *treh/treh*-like genes among 40 insect species and 31 *ath/ath-like* genes in 14 species (Table 1). Except for Dipterans, the other insect taxa have experienced specific *treh* gene duplications and maintained multiple *treh* gene copies in their genomes. Hemipteran genome showed the highest number of *treh* gene duplications (54 *treh* copies in 7 species). The pea aphid *A. pisum* possessed the highest number of *treh* gene copies with 13 *treh* genes, followed by *Aethina tumida* (Coleoptera: Nitidulidae) with 11 *treh* genes and the two aphid species *D. noxia* and *M. persicae* with 8 *treh* copies, respectively (Table 1 and Supplementary Table S1). *A. pisum* genome possesses a *treh* pseudogene with a high similarity to plant trehalases, but with a partial coding sequence due to a large deletion in the gene. *P. xylostella* possessed two *treh* genes (LOC105397091 and LOC105395616) with high level of sequence similarity with a *treh* gene encoded by *Enterobacter cloacae* (Bacteria: Enterobacteriacea; scaffold CP015227).

**Table 1:** List of species and genomes considered in the present study for the search of treh genes and transcripts.

Order	Family	Species	Annotation level of the sequenced genome	n° of *treh* genes	n° of predicted transcript variants
Diptera	Culicidae	*Aedes albopictus*	*Annotation release 101*	2	15
		*Culex quinquefasciatus*	–	2	2
	Drosophilidae	*Drosophila melanogaster*	*Release 5.30*	1	7
		*Drosophila miranda*	*Annotation release 101*	2	3
		*Drosophila pseudoobscura*	*Release 2.3*	2	3
		*Drosophila virilis*	*Release 1.2*	2	3
	Tephritidae	*Bactrocera oleae*	*Annotation release 100*	3	6
		*Bactrocera dorsalis*	*Annotation release 100*	2	2
		*Bactrocera cucurbitae*	*Annotation release 100*	2	3
		*Ceratitis capitata*	*Annotation release 101*	2	6
	Muscidae	*Musca domestica*	*Annotation release 102*	2	4
Coleoptera	Tenebrionidae	*Triboliurn castaneurn*	*Annotation release 103*	6	12
	Curculionidae	*Dendroctonus ponderosae*	*Annotation release 100*	5	13
	Nitidulidae	*Aethina tumida*	*Annotation release 100*	11	12
	Cerambycidae	*Anopiophora glabripennis*	*Annotation release 100*	7	14
Hymenoptera	Apidae	*Apis mellifera*	*Annotation release 102 Amel 4.0*	2	10
		*Apis dorsata*	*Annotation release 100*	2	2
		*Apis florea*	*Annotation release 100*	3	6
		*Bombus terrestris*	*Annotation release 101*	2	10
		*Bombus impatiens*	*Annotation release 101*	2	9
	Pteromalidae	*Nasonia vitripennis*	*Annotation release 102*	2	4
	Agaonidae	*Ceratosolen solmsi marchali*	*Annotation release 100*	2	2
	Braconldae	*Fopius arisanus*	*Annotation release 100*	3	7
		*Microplitis demolitor*	*Annotation release 101*	2	8
	Megachilidae	*Megachile rotundata*	*Annotation release 101*	2	9
	Formicidae	*Solenopsis invicta*	*Annotation release 100*	2	8
		*Acromyrmex echinator*	*Annotation release 100*	2	3
		*Camponotus floridanus*	*Annotation release 101*	2	4
		*Cerapachys biroi*	*Annotation release 101*	2	9
		*Harpegnathos saltator*	*Annotation release 101*	2	3
Lepidoptera	Bombycidae	*Bombyx rnori*	*Annotation release 101*	3	3
	Plutellidae	*Plutella xylostella*	*Annotation release 100*	6	8
	Papilionidae	*Papilio rnochaon*	*Annotation release 100*	4	4
Hemiptera	Cimicidae	*Cimex lectularius*	*Annotation release 100*	3	5
	Pentatomidae	*Halyomorpha halys*	*Annotation release 100*	8	17
	Psyllidae	*Diophorina citri*	*Annotation release 101*	6	13
	Aleyrodidae	*Bemisia tabaci*	*Annotation release 100*	9	17
	Aphididae	*Diuraphis noxio*	*Annotation releose 100*	9	12
		*Acyrthosiphon piston*	*Annotation release 101 Build 1.1*	14	23
		*Myzus persicae*	*–*	9	–

Diptera, Lepidoptera, Hymenoptera and some Hemipteran species mostly possess a unique gene coding for acid trehalase, that resulted not phylogenetically related to bacterial or fungal acid trehalase *ath* genes. Differently, multiple genes coding for acid trehalases have been identified in *A. pisum, M. persicae, P. xylostella*, and *Bactrocera oleae* (Diptera: Tephritidae) since they possessed duplicated *ath* genes as well (LOC100159015, LOC100167863, MYZPE13164_G006, MYZPE13164_G006, LOC105394851, LOC105386635 and LOC106624493, LOC106624487, LOC106622467) and *Aedes albopictus* (Diptera: Culicidae) where 11 *ath* genes have been identified (LOC109417656, LOC109417657, LOC109417661, LOC109417662, LOC109417664, LOC109622150, LOC109622151, LOC109622152, LOC109622154, LOC109622125, and LOC109622155). En exception to these previous statements is *Apis florea* (Hymenoptera: Apididae), where an *ath*-like gene LOC105737051 with high similarity to *Lactobacillus s*p. (i.e., *L. acetotolerans* AP014808.1 and *L. gasseri* CP000413.1) has been identified.

### Trehalase Phylogenetic Tree

**FIGURE 1:**
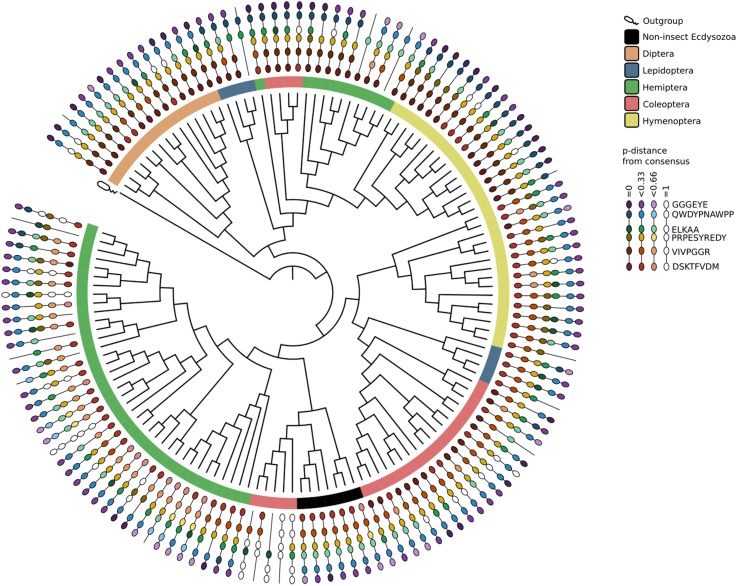
Phylogenetic tree of *treh* genes and protein isoform diversity in insects and other taxa. For each paralog only one transcript variant has been considered while protein diversity represents all the transcript variants predicted by NCBI algorithms.

Phylogenetic analysis evidenced that at least one member of the *treh-1* and *treh-2* subfamily was present in each species considered, except for Diptera that didn’t possess any *treh-1* gene (Figure 1 and Supplementary Figure S1).

*Treh-1* subfamily is represented by a higher number of members (110/160 *treh-1* genes/n° of *treh-1* and *treh-2* genes) which encode for lesser conserved trehalase isoforms, in respect to *treh-2* subfamily. Coleopteran and hemipteran *treh* genes, for instance, had species-specific duplication events involving *treh-1.* Conversely, Hymenoptera and Lepidoptera didn’t show such significant differences between *treh-1* and *treh-2*. *A. tumida* represented an interesting exception possessing unique *treh* paralogs that results more similar to trehalase genes of the outgroup species than to insects ones.

### Gene Synteny and Co-linearity

The comparison of the genomic regions containing the *treh* and *ath* genes evidenced high levels of synteny and co-linearity among species belonging to the same insect order, but not among them (Figure 7).

**FIGURE 2:**
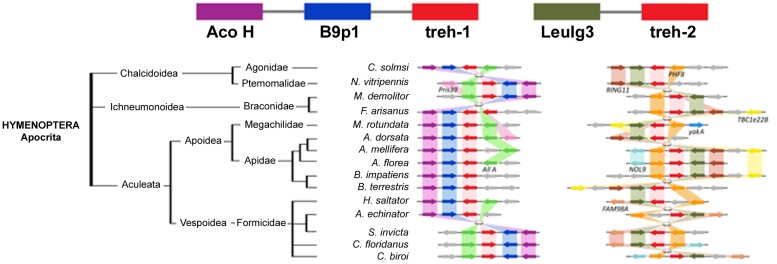
Gene synteny and co-linearity of trehalase genes in Diptera. Boxes represent syntenic genes. Arrows show reading frame of each gene. *nrnp1*, heterogeneous nuclear ribonucleoprotein L; *ZnCCHCp24*, Zinc finger CCHC domain containing protein 24-like; *uncharact*, uncharacterized protein; *U4/U6.U5*, U4/U6.U5 tri-snRNP associated protein 1; *LTV1*, LTV1 ribosome biogenesis factor; *Raba*, Rabaptin GTPase binding effector protein 1; *C3/PZPa2p8*, C3 and PZP like alpha 2 macroglobulin domain containing protein 8; *dystrob. B*, dystrobrevin beta; *PpAsx*, Polycomb protein Asx; *cof/act*, cofilin/actin de-polymerizing factor homoloug; *Chi-9*, chitinase 9; *RIP3*, ras interacting protein RIP3. Phylogenetic tree modified from *tol* (www.tol.org).

**FIGURE 3:**
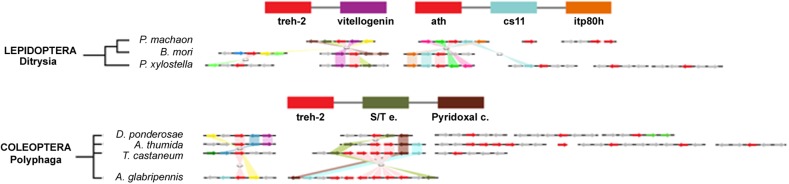
Gene synteny and co-linearity of the trehalase genes in Hymenoptera. Boxes represent syntenic genes. Arrows show the reading frame of each gene. *Aco H*, Aconitate hydratase mythocondrial like; *B9p1*, B9 domain containing protein 1 like; *LeuIg3*, Leucine-rich repeats and immunoglobulin like domains protein 3; *Pris39*, Prisilkin 39; *All A*, Allatostatin receptor like; *PHF8*, Histone-lysine demethylase PHF8 like; *RING11*, RING finger protein 11; *TBC1e22B*, TBC1 domain family member 22B; *yakA*, probable Serine/Threonine protein kinase *yakA*; *NOL9*, Polynucleotide 5^′^-hydroxyl kinase *NOL9*; *FAM98A*, FAM98A protein. Phylogenetic tree modified from *tol* (www.tol.org).

**FIGURE 4:**
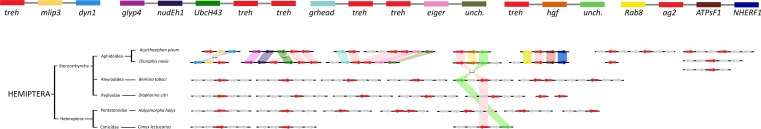
Gene synteny and co-linearity of the trehalase genes in Lepidoptera and Coleoptera. Boxes represent syntenic genes. Arrows show the reading frame of each gene. Lepidoptera; *cs11*, carbohydrate sulfotransferase 11; *itp80*, intraflagellar transport protein 80 homoloug; *DNAHQ1*, ADP dependent DNA Helicase Q1-like; *B9P1*, B9 domain containg P1; *tf Adf1*, transcription factor Adf1-like; *Tret-1*, facilitated trehalose transporter *Tret-1* like; *CatL1*, Cathepsine L1-like; *G3PDH*, glycerol 3 phosphate dehydrogenase mitochondrial; *tf Sp3*, transcription factor Sp3 or 4-like. Coleoptera; *S/T e.*, Ser/Threo extracellular; *Pyridoxal c.*, pyridoxal carboxylase; *Ser pst*, Serine proteinase stubble; *Rho190*, Rho GTPase active protein 190; *Tsk2, Trans*-skeletase 2; *PlexA*, Plexine A; *Ub c.H*, Ubiquitin carboxyl hydrolase. Phylogenetic tree modified from *tol* (www.tol.org).

The highest level of genomic synteny was found in Hymenoptera with 3 *treh* neighboring genes shared by 15 species on 30 genomic scaffolds (Figure 2), Coleoptera (Figure 3), and Lepidoptera (Figure 3). Hemipterans, on the opposite, show very low synteny with no *treh* neighboring genes shared by all species studied (Figure 4). However, within the super-family Aphidoidea, 13 shared *treh* neighboring genes have been found between *A. pisum* and *D. noxia*, considering 44 genomic scaffolds and *54 treh* genes (Figure 4).

**FIGURE 5:**
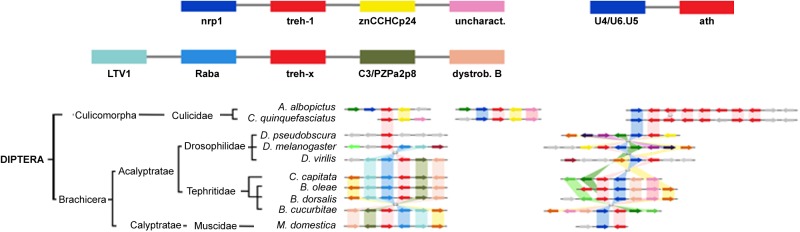
Gene synteny and co-linearity of the trehalase genes in Hemiptera. Boxes represent syntenic genes. Arrows show reading direction of each gene. *mlip3*, mucolipin 3; *dyn1*, dynamin 1 like protein; *glyp4*, glypican 4 like; *nudEh1*, nuclear distribution protein nudE homolog 1; *UbcH43*, Ubiquitin carboxyl-terminal hydrolase 43; *grhead*, protein grainyhead; *eiger*, protein eiger; *unch.*, uncharacterized; *hgf*, hepatocyte growth factor-regulated tyrosine kinase substrate; *Rab8*, ras related protein Rab 8A; *ag2*, alpha-glucosidase 2; *ATPsF1*, ATP synthase mitochondrial F1 complex assembly factor 1; *NHERF1*, Na (+) / H (+) exchange regulatory cofactor NHE-RF1. Phylogenetic tree modified from *tol* (www.tol.org).

*Aedes albopictus* and *Culex quinquefasciatus* (Diptera: Cucilidae) duplicated the entire region harboring *treh* genes and lack of synteny with Diptera Brachicera scaffolds (Figure 5).

### Paralogs With Protein Functional Specialization

**FIGURE 6:**
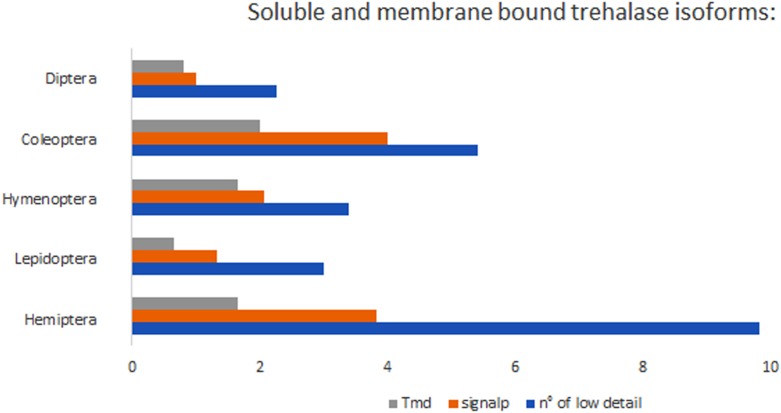
Soluble (orange) and membrane bound (gray) trehalase isoforms in different insect orders.

To evaluate the solubility of trehalase and acid trehalase enzymes, the predicted protein sequences were tested with *Signal p 4.1* to identify potential signal peptides and *TMHMM Server v. 2.0* to predict transmembrane domains (Figure 6).

**FIGURE 7:**
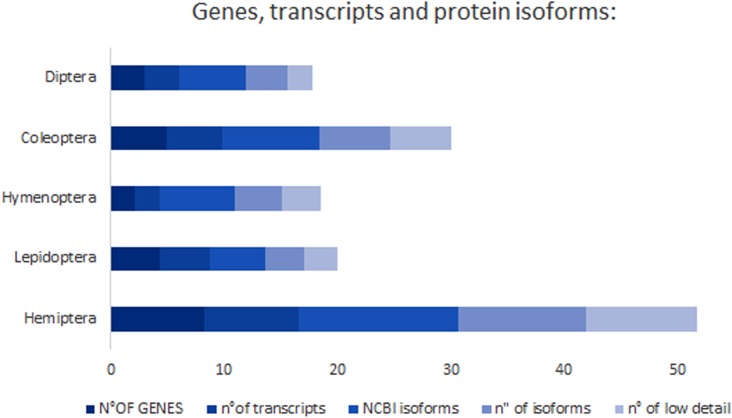
Number of genes, transcript variants and protein isoforms in the insect species studied.

In order to test if *treh* gene duplications were accompanied by functional divergence, the number of genes, transcript variants and protein isoforms has been compared among insect species (Figure 7). Isoform diversity has been analyzed firstly considering complete protein amino acid sequences and their conservation at the whole sequence level (isoforms *sensu stricto*) and secondly focusing only on the conservation of the amino acidic sequence of the functional domains occurring in these proteins (isoforms *sensu lato*).

The number of protein isoforms varied widely and reached the highest rates in Coleoptera (2,687 isoforms *sensu stricto*/n° of species; 1,6875 isoforms *sensu lato*/n° of species) and Hemiptera (2,020 isoforms *sensu stricto*/n° of species; 1,405 isoforms *sensu lato*/n° of species). The lowest diversity can be found in Hymenoptera with 0,444 isoforms *sensu stricto*/n° of genes and 0,227 isoforms “*sensu lato*”/n° genes (Table 2). *A. thumida* possess high isoform diversity representing an interesting exception in Coleoptera (Table 2).

**Table 2:** Distribution of trehalase genes in insects, evaluating for each Order the number of genes (g), transcripts (t), isoforms, isoelectric point, molecular weight (MW), size (expressed as number of amino acids, aa), solubility and presence of transmembrane domains (tm).

	n° species	n° of genes	n° of transcripts	t/g	n° isoforms S	n° isoforms L	genes/species	transcripts/genes	isoforms S/species	isoforms l/species
*Hemiptera*	7	54	93	1,722222222	14,14285714	9,833333333	7,714285714	1,722222222	2,020408163	1,404761905
*Lepidoptera*	3	9	11	1,222222222	5	3	3	1,222222222	1,666666667	1
*Hymenoptera*	15	32	93	2,90625	6,666666667	3,4	2,133333333	2,90625	0,444444444	0,226666667
*Coleoptera*	4	29	51	1,75862069	10,75	6,75	7,25	1,75862069	2,6875	1,6875
*Diptera*	11	36	49	1,361111111	5,909090909	2,272727273	3,272727273	1,361111111	0,537190083	0,20661157

	**pIS**	**MW S**	**aa number S**	**% sol**	**% tm**					

*Hemiptera*	6,314	62624,89	531,91	47,42268041	26,80412371					
*Lepidoptera*	5,746	71783,09	621,8	40	33,33333333					
*Hymenoptero*	5,935	74914,02	638,35	63,82978723	69,14893617					
*Coleoptero*	6,048	64650,7	547,98	76,47058824	29,41176471					
*Diptero*	5,276	69969,51	619,78	40	44,61538462					

Hemiptera trehalase isoforms showed a higher average isoelectric point (6,314), a lower average molecular weight (62624,89 g/mol) and amino acid number (531,91) than other insects, but no significant differences have be noticed evaluating soluble isoforms percentage (47,42% of isoforms possess a signal peptide in aphids) or the presence of transmembrane domains (26,80% of isoforms has at least one transmembrane domain) (Figure 6, 7 and Table 2).

All acid trehalases genes, on the contrary, encoded for only one isoform, apparently lacking exons and introns. Amino acidic sequences of predicted ATH isoforms were conserved only within insect orders and never possessed typical bacterial or fungal *ath* domains (Table 2).

## Discussion

### *Treh* Genes Duplication and Trehalase Sub-Functionalization in Insects

Trehalose is commonly present in the haemolymph of most insects and it has been suggested a role of this sugar in osmoregulation in some insects (Iturriaga et al., 2009).

The analysis of the evolutionary history of the *treh/treh*-like and *ath/ath*-like genes and their functional divergence during the insect evolution evidenced that at least two *treh* paralogs are present in most of studied species, except for Dipterans, that probably never duplicated the *treh* gene. Many insect species have experienced specific *treh* gene duplications and maintained the multiple *treh* genes as functional copies (paralogs) in their genomes (Avonce et al., 2006; Tang et al., 2018).

This is interesting since, according to literature (Zhang, 2003; Zhang et al., 1998), a loss of function generally occurs for most of the paralogs. The *treh* gene family in insects represents therefore an interesting exception for studying the adaptive effect of duplications. Gene duplication is a major evolutionary mechanism that can confer adaptive advantages to organisms through the occurrence of mutations in paralogs resulting in new genetic variants (Duda and Palumbi, 1999; Lynch and Conery, 2003; Conant and Wolfe, 2008; Warren et al., 2014). Indeed, paralogous genes may have a decreased purifying selective pressure resulting in the fixation of mutations. In some rare cases, no deleterious mutations occur so that paralogs are maintained functionally active and may undergo processes of sub-functionalization (with paralogous and orthologous genes cooperating to the same function) or a neo-functionalization, based on the gaining of new functions of paralogous genes in respect to orthologous ones (Hughes, 1994; Zhang et al., 1998; Force et al., 1999; Kellogg, 2003; Zhang, 2003; Innan and Kondrashov, 2010).

The presence of low sequence conservation at C and N-termini of the amino acidic sequences of trehalase isoforms in Hemiptera and Coleoptera, in comparison to other insects, suggests that a functional divergence occurred in *treh* family during the evolution of these taxa. Interestingly, most of the Coleopteran *treh* gene duplications involved the *treh-1* gene only and paralogs are clustered in the same scaffold. The presence of multiple copies of the *treh* genes in Coleoptera could be explained as an adaptation to a trehalose rich diet in insectivorous, detritivorous, and mycophagous species since this sugar is present at high concentration in fungi (Thevelein, 1984). *A. thumida*, on the contrary, represents an exception since, despite its ecological adaptation as a beehive parasite, it possesses a higher number of *treh* copies encoding for a high number of trehalase isoforms. In *P. xylostella*, the finding of trehalase similar to *E. cloacae* *treh* probably derives from a bacterial DNA contamination of genomic database considering that *E. cloacae* is widely adopted in agriculture as a bio-control agent against pathogens (Duponnois et al., 1999; Watanabe et al., 2001).

Trehalase genes have been duplicated more frequently in Hemipterans than in other insects, but numerous rearrangements (including inversion and conspicuous genomic insertions or deletions) seem to have occurred in their genomes so that the multiple *treh* genes were not clustered in the same scaffold. This result is not surprising considering the holocentric nature of their chromosomes that can confer the ability to retain chromosomal rearrangements, such as intrachromosomal translocations and/or chromosomal fission/fusions (Manicardi et al., 2015). Furthermore, hemipteran trehalases have the longest glycine-rich domain and the higher rate of fixed mutations. Both these aspects seem to be functionally relevant since the additional amino acids enriched in the glycine rich regions are likely to influence the interactions of these regions with other proteins or RNA and may facilitate homo- and hetero-meric interactions (Wang et al., 1997; Gsponer et al., 2008). The high diversification of *treh* gene family in Hemipterans is particularly interesting, since it suggests that the presence of multiple copies of these enzymes is not the result of an adaptation to a sugar-rich diet (that should favor the presence of multiple copies of highly similar genes), but could be due to the occurrence of different roles of trehalase in these species. Indeed, according to literature data, defective or inhibited trehalases may be associated in insects to altered sugar metabolism (Wegener et al., 2003) or to morphological abnormalities (Zhang et al., 2012) suggesting that *treh* gene duplication could result in a sub-functionalization of trehalases in Hemiptera.

The role of acid trehalases in insects is still unknown and horizontal gene transfer events from bacteria or fungi to insects could be involved. Horizontal gene transfer has been indeed already suggested, for instance, to explain the presence of carotenoids genes in aphids (Moran and Jarvik, 2010; Mandrioli et al., 2016) and the occurrence of seven highly expressed trehalase genes with strong similarity for bacterial trehalases in the rotifer *Adineta vaga* (Hespeels et al., 2015). However, insect acid trehalases were not phylogenetically related to bacterial and fungal *ath* and they don’t possess the functional domains typically observed in the ATH proteins so that a different origin of these genes should be evaluated. Differently, the presence of bacterial *ath*-like genes in *A. florea* seems to result from a bacterial DNA contamination of genomic database since *Lactobacillus* sp. is reported as a typical component of the *A. florea* microbiota (Saraithong et al., 2014).

### Trehalase Diversification in Response to Plant Inhibitors: Salivary Proteins as Key Molecules in the Co-evolution of Aphids and Their Host Plants

Trehalase enzymes play important roles in the insect metabolism so that they are related to the insect survival (Iturriaga et al., 2009). For this reason, in phloem sap sucking insects, trehalase enzymes represent molecules that host plants can target to establish efficient defensive strategies. For instance, plants produce trehalose as a signal molecule in response to aphid infestation (Smith and Boyko, 2007; Louis et al., 2012) and the presence of trehalase in aphid saliva may be relevant to modulate the trehalose-based defensive plant pathways (Cooper et al., 2010, 2011; Cui et al., 2012; Vandermoten et al., 2013; Chaudhary et al., 2015). In particular, trehalases in aphid saliva could act as PAD4 suppressor blocking the local accumulation of trehalose in the wounded plant tissue (Singh and Shah, 2012; Bansal et al., 2013).

At the same time, however, plants evolved in their turn trehalase inhibitors (Tatun et al., 2014) in a true arms race against phytophagous insects resulting in a strong selective pressure on the *treh* gene family that resulted in the maintenance of duplicated *treh* copies and in their divergence in order to allow aphids and other sap sucking insects to escape the plant defensive strategies.

The presence of co-evolution between plant trehalase inhibition and duplication/fixation of mutations in the *treh* genes could be particularly relevant in aphids in view of their peculiar reproductive mode. Indeed, the reproduction of aphids is mainly based (with the exception of a unique generation in autumn) on apomictic parthenogenesis consisting in several thelytokous parthenogenetic generations, in which unfertilized eggs develop into females (Nardelli et al., 2017). In the absence of an amphygonic reproduction, aphids cannot have any recombination between female and male genomes during spring and summer causing a reduced gene flow that will delay the spread of advantageous alleles. In this case, this means that *treh* alleles with favorable mutations couldn’t be spread in aphid populations during the parthenogenetic phase of their life cycle. Interestingly, the occurrence of multiple copies of the *treh* genes within the aphid genomes could allow the presence of multiple alleles in the same genome making gene duplication and mutations a sort of alternative pathway (in respect to genome recombination) to favor the presence of advantageous alleles.

Aphids seems to be particularly unusual in term of presence of duplications since they possess four times the gene duplications observed on average in other arthropods (The International Aphid Genomics Consortium, 2010; Mathers et al., 2017), a feature that is in common, together with the reproduction based on parthenogenesis, with the water flea *Daphnia pulex* (Crustacea: Cladocera). In aphids and *D. pulex* most of identified duplications are clade-specific and it has been suggested that duplicates were involved in rapid adaptation to environment (Pennisi, 2009; Simon et al., 2011). From this view, *treh* gene duplications in aphid could be an effective tool in a molecular adaptive strategy evolved to adapt to host plants, in absence of sexual reproduction and allelic recombination. The *treh* gene family has been indeed involved in many aphid-specific duplication events and *treh* paralogous genes (possessing different mutations) could be retained to face the evolution of plant trehalase inhibitors in a sort of aphid-host plant arm race (Shcherbakov and Wegierek, 1991; Hong et al., 2009; Szwedo and Nel, 2011; Liu et al., 2014).

In view of the relevant role that *treh* genes could play in aphids, the understanding of the biochemical nature and physiological function of trehalases could be therefore useful not only from an evolutionary point of view, but also at an applicative level, since a better understanding of trehalase could be crucial to develop new insectcides (based on trehalase inhibitors) or plant cultivars more resistant to aphids and/or to other sap sucking agricultural pest insects.

## Author Contributions

All the authors contributed to the data analysis and interpretation, drafting and revising the manuscript, and approved the final version of the manuscript. The original study design was made by AN and discussed with the other authors.

## Conflict of Interest Statement

The authors declare that the research was conducted in the absence of any commercial or financial relationships that could be construed as a potential conflict of interest.

## Footnotes

1 https://www.ncbi.nlm.nih.gov/genbank/

2 http://bipaa.genouest.org/is/aphidbase/

## Supplementary Material

FIGURE S1 Phylogenetic distribution of the identified treh genes.

TABLE S1 Complete list of the identified treh genes and protein main features.
