# Supplementary figures and images for: The Evolutionary History and Functional Divergence of Trehalase (treh) Genes in Insects
Source: Front Physiol. 2019 Feb 15;10:62. doi: 10.3389/fphys.2019.00062 (PMC6384254; doi:10.3389/fphys.2019.00062)

# Dataset\_legend

value1

value2

value3

value4

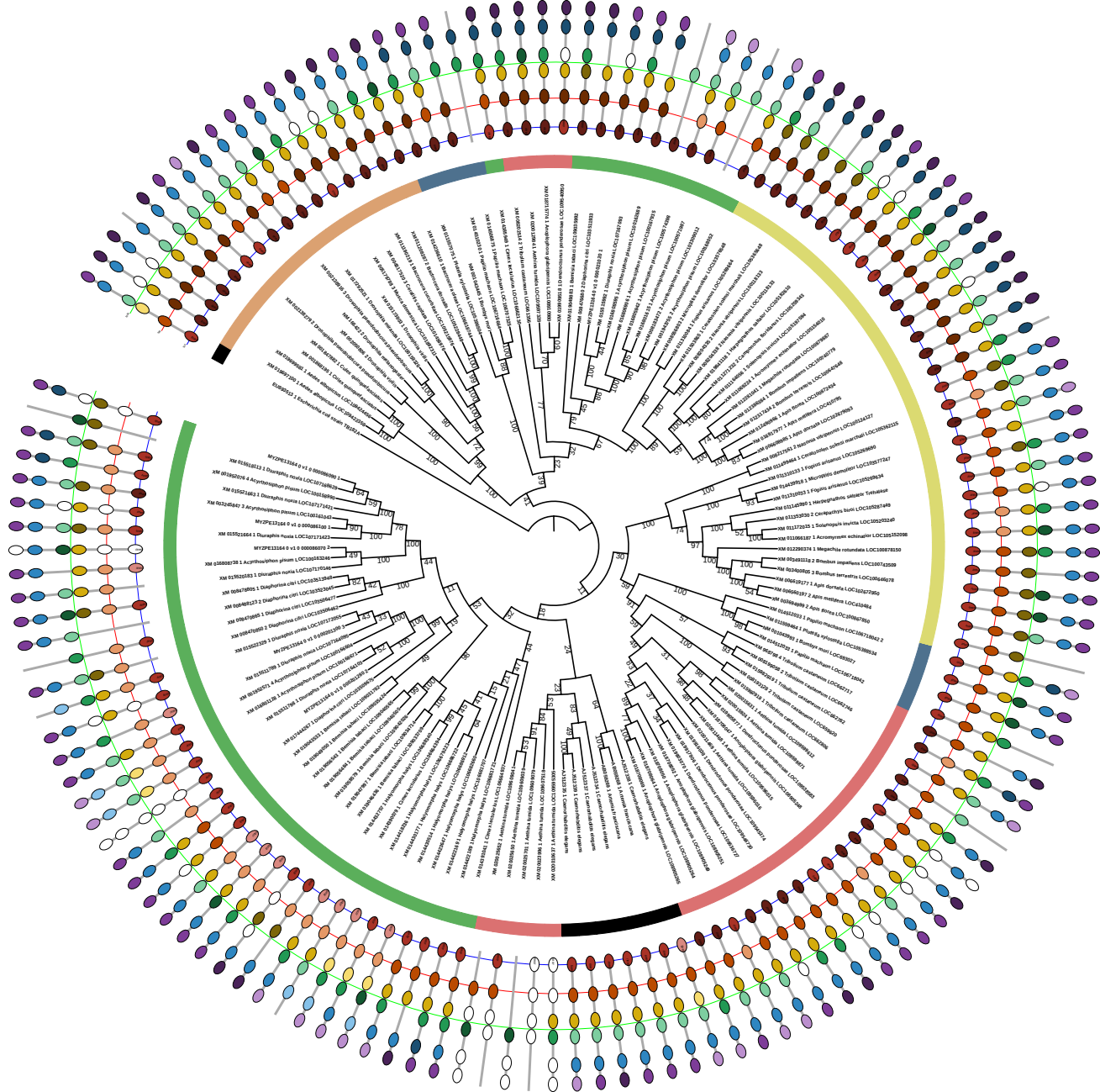

Supplement: TABLE S1 — Complete list of the identified treh genes and protein main features. [file Data_Sheet_1.PDF]
